# Dealing with tobacco use and dependence within primary health care: time for action

**DOI:** 10.1186/1617-9625-11-6

**Published:** 2013-02-26

**Authors:** Constantine Ilias Vardavas, Emmanouil K Symvoulakis, Christos Lionis

**Affiliations:** 1 Department of Social Medicine, Clinic of Social and Family Medicine, University of Crete, Heraklion, Greece; 2 Department of Social and Behavioral Sciences, Center for Global Tobacco Control, Harvard School of Public Health, Boston, USA

## Main text

Primary health care has an operational advantage in overall health promotion, as preventive activities and the management of multimorbidity are placed highly within its agenda. A key component of health promotion is tobacco prevention and smoking cessation, which in the developed world, is the largest preventable cause of death and disability, and estimated to cause 6 million preventable and premature deaths every year [1]. With the above dire number in mind, the World Health Organization has called for smoking cessation to be integrated into primary health care globally [2,3].

Patient centered primary care is seen as the most suitable health system “environment” for providing advice on smoking cessation and general practitioners/family physicians thus have a framework to advocate for smoking cessation within their daily practice [4]. Based on this framework, guidelines have been issued and promoted within primary care practice [4,5]. The first step in handling tobacco control and smoking cessation within daily clinical care is to identify smokers a practice has been shown to increase the likelihood of smokers being provided with help to quit [5], and provide primary care practitioners the chance to refer smokers to a cessation program [6]. Indeed, within the UK, 73% of new patients within primary care were reported to have their smoking status recorded within 90 days of registering - an indicator of smoker engagement- with 44% of these entries being recorded on the day of registration [7] The above numbers, although promising, are still far from those that primary care can potentially achieve. Literature frequently reports failure to advise on smoking cessation during clinical visits and this opportunity may be largely missed by clinicians, especially in developing countries or in countries under fiscal constraints [8]. This gap in engagement has been identified amongst others to be associated with the lack of official training in tobacco control during undergraduate years or residency [9], the lack of time due to organizational constraints and increased work load [10], the smoking habits of the primary care provider [11], or the lack of appropriate social-cognitive training [12].

While understaffing, work load and the lack of time with each patient may constrain the ability to fully develop smoking cessation programmes, screening for nicotine dependence and potential relapse among patients can be performed by nurses, health care workers and psychologists as part of the multidisciplinary team [13]. This integrated primary health care team can subsequently engage smokers or refer them to a more specialized smoking cessation clinic, which makes their potential involvement furthermore promising [14]. Research has indicated that smoking cessation treatments applied by non-specialists, may be equally effective in primary care [15]. Thought vigilant patient follow up, primary care providers can also support abstinence with success, as complex interventions support annual abstinence rates of 30% with shorter follow up periods indicating even higher rates [14,16]. However, to obtain such elevated abstinence rates, behavior modification related skills, such as extended cognitive behavioral therapy must be acquired by the primary health care team [17]. Indeed, cognitive behavioral therapy has been identified for example to be able to provide specific benefits to smokers with a history of recurrent depression, and has been identified as a potential reason for lack of engagement [12,18].

Primary care, endorsing the concept of patient centeredness, can offer the opportunity to include smoking cessation as a prerequisite action of managing multimorbidity and mental health disorders [19]. As most patients with multi-morbidities are actually seen in primary health care practice [20], with a substantial percentage of patients known to have established multiple comorbidities that can be associated with smoking, such as COPD or mental illness [9,21-24], the need for the adoption for a patient centered approach unfolds as an imperative action. Such patient centered care, although initially introduced in its humanistic dimension by Balint in 1969 [25] has since then been multilaterally focused on its bio-psychosocial direction, understanding that the patient is a person that shares both the power and the responsibility with the health care provider with regards to their management and health related decisions [26]. This joint responsibility between the health care provider and the patient, is a key aspect of handling smoking cessation within a community based setting, as it identifies the need for teamwork and the understanding of the smokers concerns and needs during this fragile transitional phase in the smokers life.

As the economic crisis that has emerged recently may both limit the access and the utilization of health care services [27,28] and increase the populations susceptibility to develop substance abuse and/or mental health disorders, it is important that each opportunity for intervention is utilized [29]. To that direction, smoking prevention and cessation should and can be integrated into daily clinical practice services provided by a primary care practitioner capable to manage the patients’ multimorbidity. As tools exist and skills can be empowered-evidence has indicated that it can be done very quickly based on the “5 As” [30,31] - all that is needed now is action. Conclusively, screening for tobacco use in clinical practice is as simple as asking the patient “***Do you currently smoke cigarettes or use any other tobacco product?***”
